# Endoscopic submucosal dissection of a gigantic gastric polyp aided by a novel retraction device and complicated by upper esophageal sphincter laceration during retrieval

**DOI:** 10.1016/j.vgie.2022.10.014

**Published:** 2022-12-02

**Authors:** Michael Lajin, Fateh Bazerbachi, Octavio Armas

**Affiliations:** 1 Sharp HealthCare, San Diego, California; 2 CentraCare, St. Cloud, Minnesota

**Keywords:** ESD, endoscopic submucosal dissection

## Abstract

Video 1 Endoscopic submucosal dissection of a sizeable gastric polyp with a short and broad stalk assisted by a novel traction device.

Endoscopic submucosal dissection of a sizeable gastric polyp with a short and broad stalk assisted by a novel traction device.

## Case

**Figure 1:**
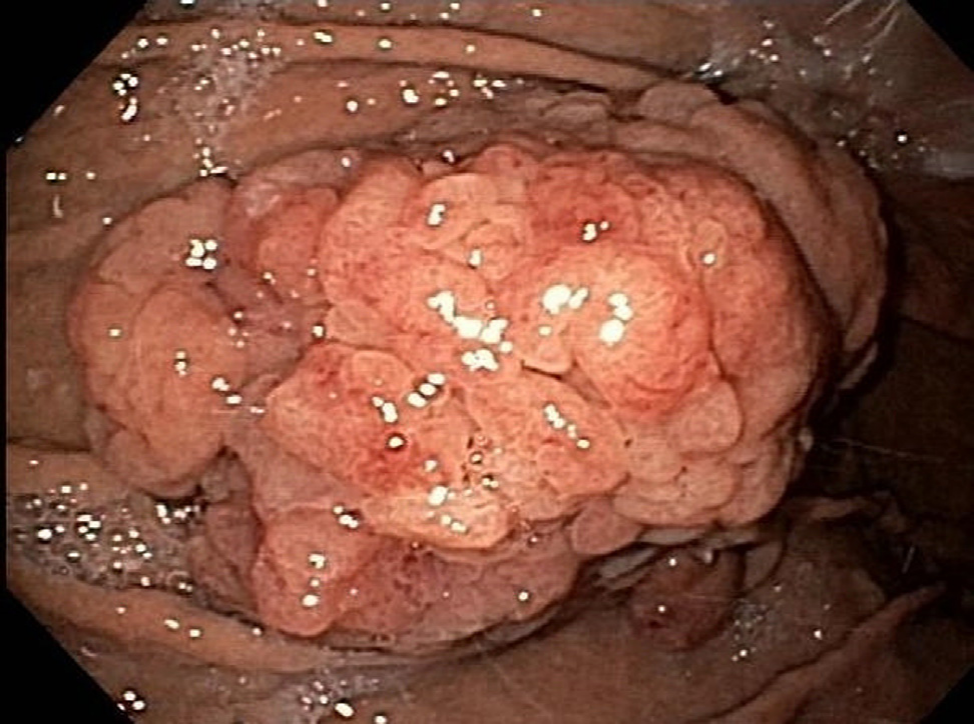
Endoscopic image of the large adenomatous gastric polyp.

**Figure 2:**
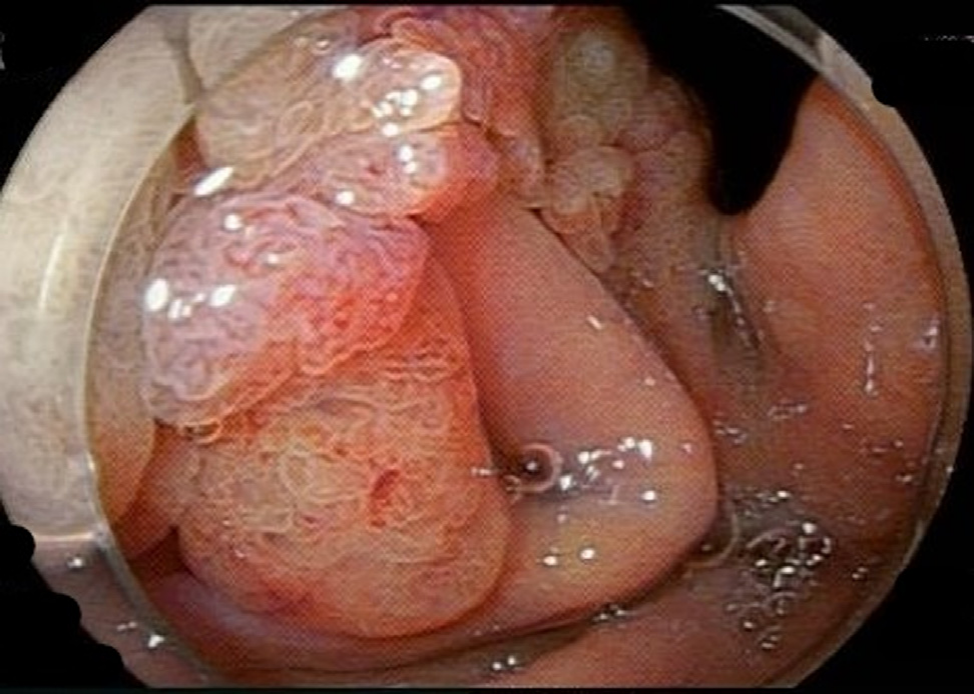
Endoscopic image of the short and broad stalk.

An 87-year-old woman with chronic renal insufficiency and coronary artery disease on clopidogrel was found to have a sizable adenomatous gastric polyp. She was referred for endoscopic resection after declining surgery. On endoscopy, a gigantic polypoid lesion was found measuring 11 × 8 cm (Fig. 1) with a broad short stalk (Fig. 2). The surface pattern was irregular without ulceration. For the most part, the vascular pattern was a mesh of well-connected vessels. However, it contained an area of irregular vascular patterns worrisome for high-grade dysplasia. An EUS showed no invasion through the gastric wall, lymphadenopathy, or liver lesions.

## Procedure

Attempting polypectomy using an endoloop was challenging because of the size and the short stalk.^1^ High-grade dysplasia was suspected, and the decision was made to resect it en bloc by endoscopic submucosal dissection (ESD).^2^, ^3^, ^4^ The ESD technique also allows for coagulation of encountered blood vessels, a characteristic feature of polyps with a broad stalk. Because it was difficult to expose the different sides of this short and broad stalk, we decided to use a novel retraction device (Tracmotion; Fujifilm, Tokyo, Japan). Tracmotion is a single-operator, retraction device. It has a 360-degree rotatable jaw. This enables manipulating the lesion in different directions to expose the stalk, which in turn facilitates performing a circumferential mucosal incision on the stalk. The device also provides traction and exposes the submucosa to facilitate submucosal dissection. The device remains steady without a locking mechanism providing a stable position during ESD and freeing the hand of the operator to manipulate the shaft of the endoscope. It requires dual-channel endoscopes with at least 3.7-mm channels.

**Figure 3:**
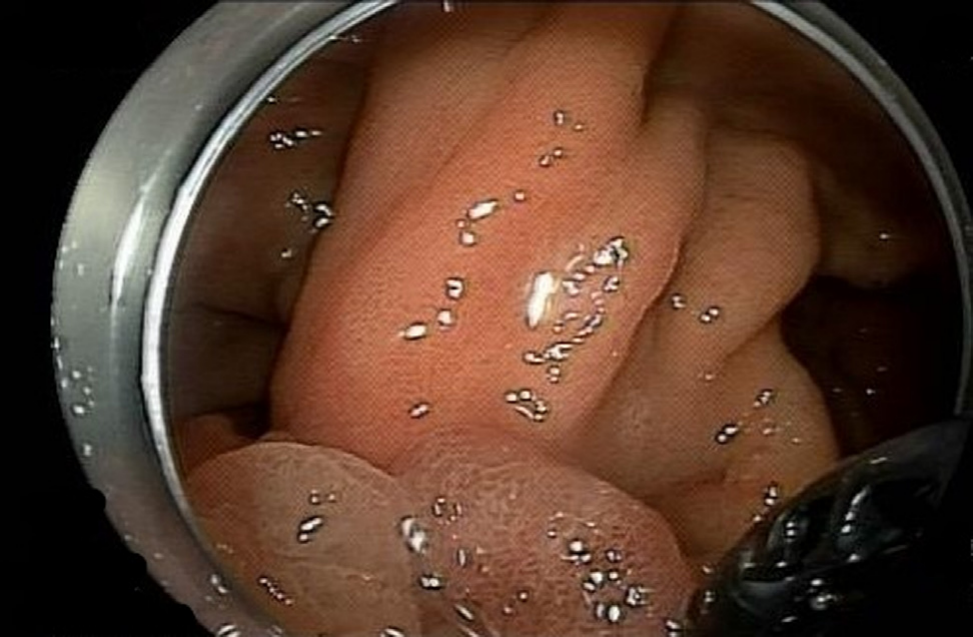
Exposing the stalk of the polyp using the retraction device.

**Figure 4:**
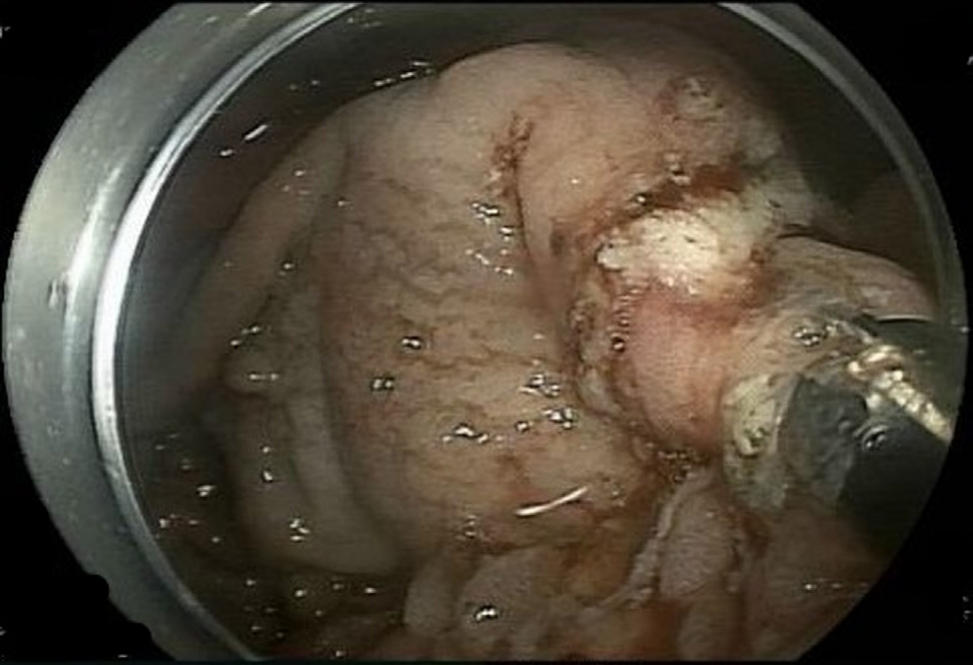
Using the retraction device to facilitate the circumferential mucosal incision at the stalk.

**Figure 5:**
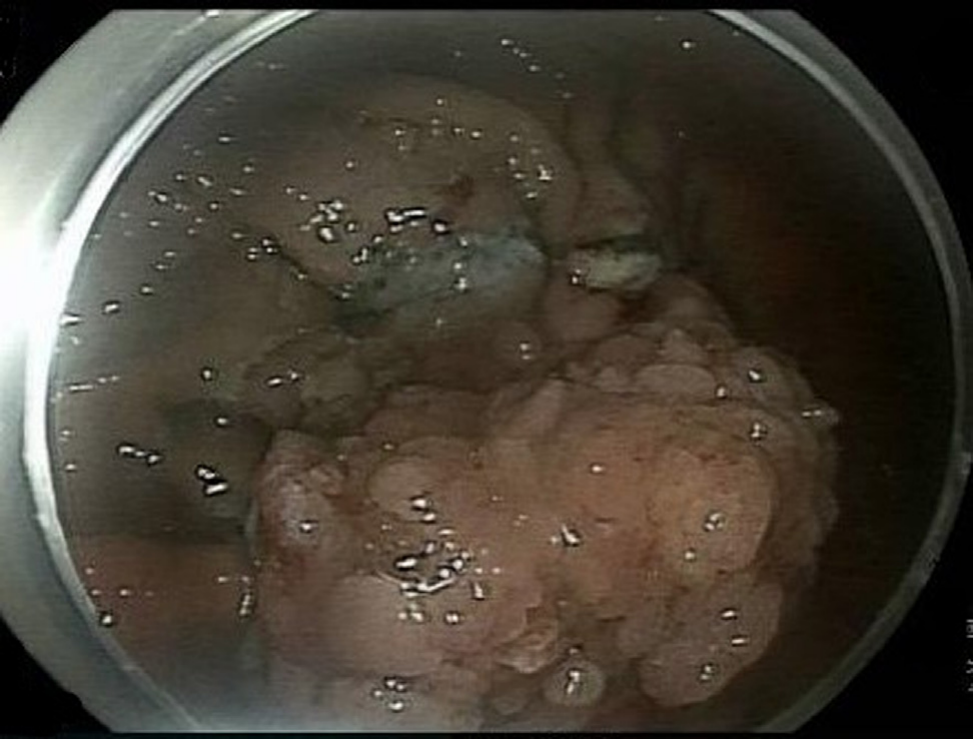
A near-circumferential mucosal incision at the stalk is completed.

**Figure 6:**
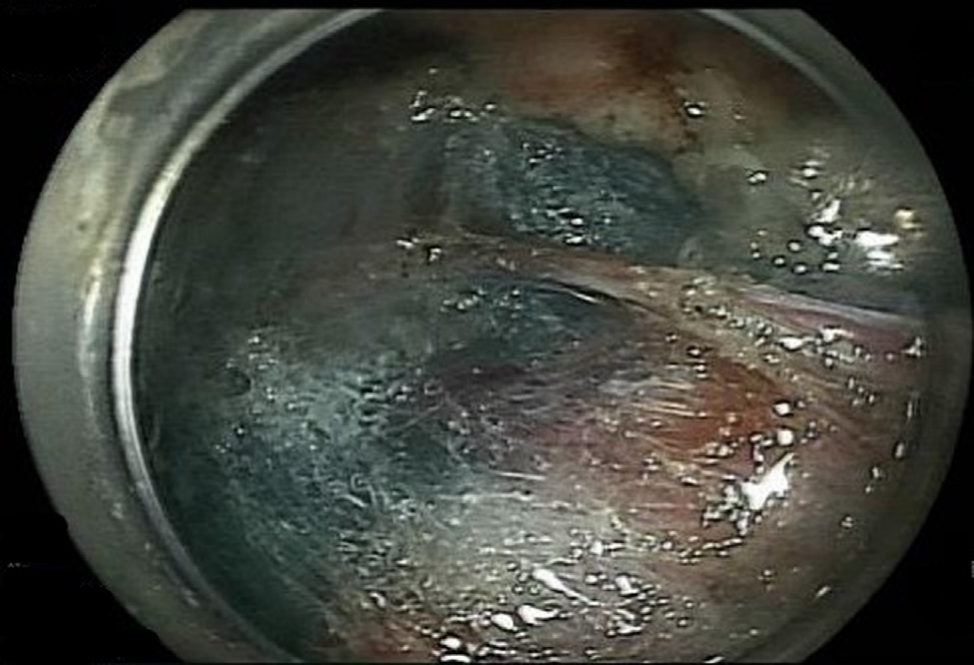
Exposed submucosal fibers and blood vessels at the stalk.

Using this device, we exposed the different aspects of the stalk (Fig. 3), allowing submucosal injection (normal saline, methylene blue, and epinephrine) followed by a near-circumferential mucosal incision (HybridKnife, Endo Cut; ERBE, Marietta, Ga, USA) at the stalk (Figs. 4 and 5). Submucosal dissection was then performed (HybridKnife: preciseSECT, Olympus, Tokyo, Japan). Encountered blood vessels (Fig. 6) were coagulated with the same needle for small blood vessels and with coagulation forceps (Olympus, Tokyo, Japan) for larger blood vessels.

**Figure 7:**
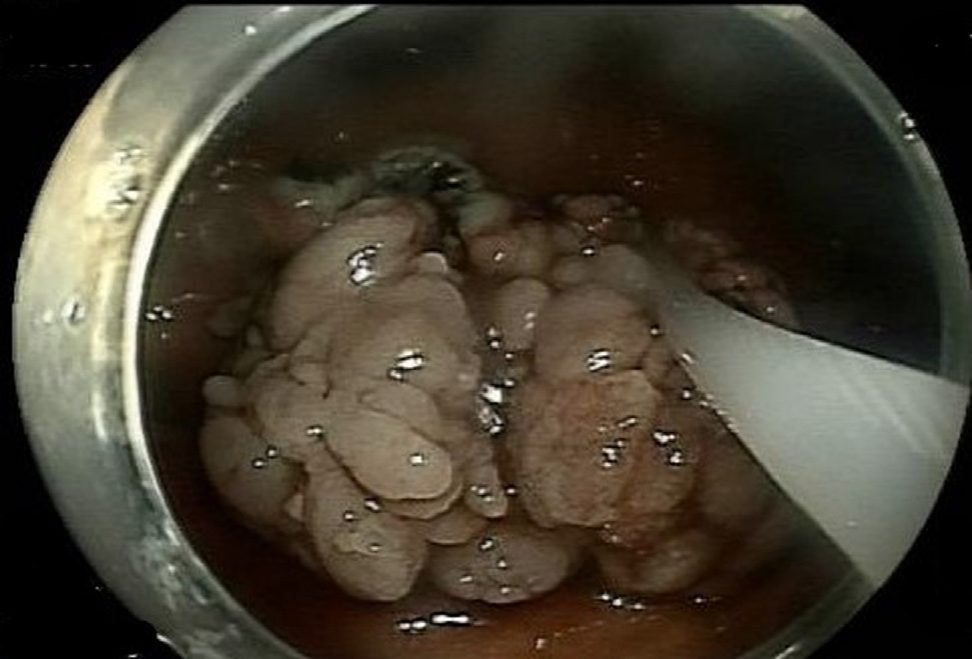
En bloc resection of the polyp is completed.

**Figure 8:**
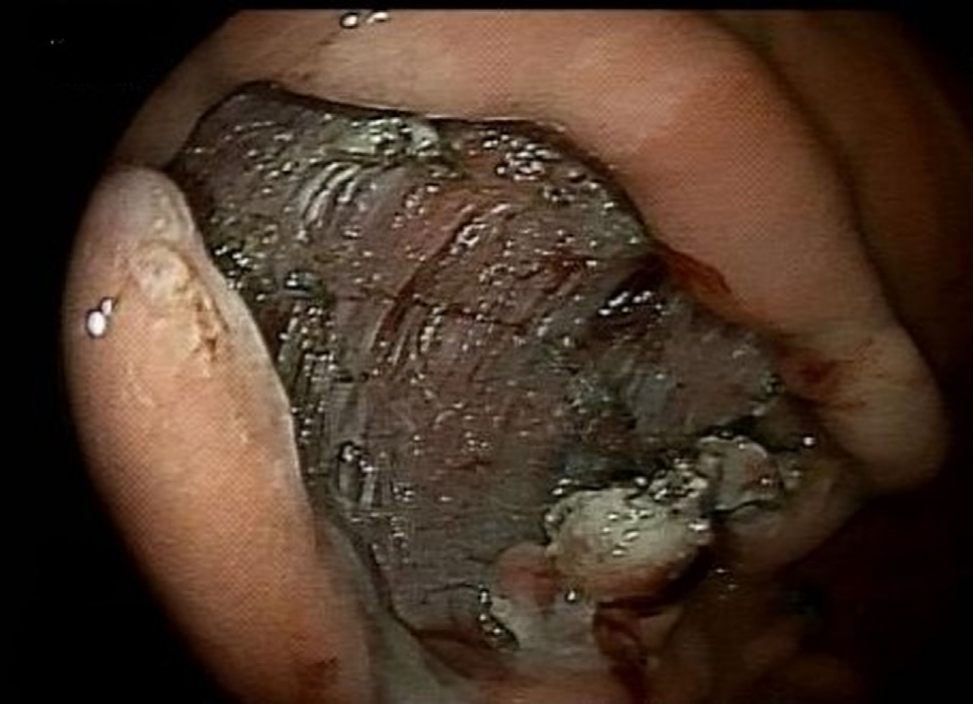
The postendoscopic submucosal dissection defect.

After completing the submucosal dissection, the remaining mucosal bridge was cut using an insulated insulation-tipped knife to avoid injury to the gastric wall behind the lesion. The lesion was resected en bloc (Fig. 7) without adverse events. However, the large size of the lesion was prohibitive for en bloc retrieval. The lesion was fragmented and retrieved one piece at a time. The ESD defect (Fig. 8) was closed with clips.

## Outcome

Despite fragmentation, retrieval resulted in a deep tear at the level of the upper esophageal sphincter resulting in subcutaneous emphysema without clinical features of mediastinitis. The patient was managed conservatively with parenteral antibiotics, bowel rest, and parenteral nutrition. No surgery was required. The subcutaneous emphysema resolved after 72 hours. An esophagram was performed after 2 weeks and showed complete healing. The final pathology showed adenoma with high-grade dysplasia and intramucosal cancer. The margins of the high-grade dysplasia were clear. No invasion of the polyp stalk or submucosa was identified in any of the fragments. A follow-up endoscopy after 4 months showed a scar without evidence of residual adenoma or malignancy (Video 1, available online at www.giejournal.org).

## Conclusions

Careful inspection and characterization of large adenomatous gastric polyps are vital in determining the best resection plan. Additionally, ESD enables en bloc endoscopic resection of gigantic gastric polyps with broad and short stalks. In this setting, a rotatable retraction device can be used to expose the different sides of the stalk to facilitate the circumferential mucosal incision. Last, because the stalk is the most important piece for histological evaluation, one possibility of retrieving this large gastric lesion after en bloc resection is to incise and retrieve the entire stalk in one piece and then fragment the rest of the lesion into smaller easily retrievable fragments.

## Disclosure

*All authors disclosed no financial relationships.*

## Supplementary data

Video 1 Endoscopic submucosal dissection of a sizeable gastric polyp with a short and broad stalk assisted by a novel traction device.
